# Multilevel bivariate analysis of the association between high-risk fertility behaviors of birth and stunting with associated risk factors in Ethiopia

**DOI:** 10.3389/fnut.2024.1355808

**Published:** 2024-05-31

**Authors:** Wondaya Fenta, Melkamu A. Zeru

**Affiliations:** Department of Statistics, College of Science, Bahir Dar University, Bahir Dar, Ethiopia

**Keywords:** high-risk fertility behavior, stunting, Ethiopian, multilevel, bivariate

## Abstract

**Introduction:**

Currently, the linkage between high-risk fertility behavior of birth and the occurrence of stunting among children under the age of 5 continues to be a significant public health problem in developing countries, including Ethiopia. This issue poses a threat to the health and overall wellbeing of under-five children. Thus, the main objective of this study was to examine the association between high-risk fertility behavior of birth and the stunting status of children and associated factors.

**Methods:**

The data used for this study were extracted from the recent Ethiopian Mini Demographic and Health Survey data in 2019. A total weighted sample of 4,969 under-five children was included in this study, and the relevant data were extracted from those samples. The multilevel bivariate analysis was used to assess the association between high-risk fertility behavior of birth and the stunting status of under-five children in Ethiopia.

**Results:**

It was found that, out of 4,997 under-five children, 24% of under-five children experienced stunting as a result of high-risk fertility behavior of birth. Our study also revealed an intra-class correlation of 0.2, indicating that 20% of the variability in both high-risk fertility behaviors of birth and stunting can be attributed to differences between communities. Furthermore, there was a statistically significant association between high-risk fertility behavior of birth and the stunting status of children under the age of 5 years [AOR = 8.5, 95% CI: (5.58, 18.70)]. Similarly, the stunting status of birth among boys was 1.36 times greater than the estimated odds of the stunting status of birth among girls [AOR = 1.36, 95% CI: (1.19, 1.55)].

**Conclusion:**

This study found that there was a significant statistical association between high-risk fertility behavior of birth and stunting status of under-five children. Specifically, children born to mothers under 18 years and in households with high parity were identified as the main risk factors for child stunting. Furthermore, health-related education, improved access to maternal healthcare, and training interventions were associated with high-risk fertility behavior during birth and child stunting. The study suggests that regular health assessments and early interventions for infants born to mothers with high-risk reproductive characteristics are crucial to reducing the impact of child stunting under 5 years of age.

## Introduction

Childhood stunting is one of the most significant health problems that should not be ignored in the public health realm (1). It is further indicated as the irreversible outcome of inadequate nutrition and a major cause of morbidity during the first 1,000 days of a child’s life (2–4). Among various risk factors of childhood stunting, mother’s age at the time of birth, inter-pregnancy interval, and child’s birth order have particularly been highlighted as some of the most critical risk factors behind childhood stunting (5, 6).

Globally, in 2020, approximately 149 million children under the age of 5 were stunted (7). Notably, Asia contributed 87 million stunting, and Africa is sharing 59 million to the burden of stunting. Even though the total fertility rate is declining globally, it is decreasing more slowly in Sub-Saharan Africa, with a total fertility rate of 4.69 children per woman in 2018, down from 5.37 in 2008 (8). Despite declining natural resources, a lack of infrastructures, such as housing, schools, and health facilities, and increased chronic under nutrition of birth, most African countries lack a demography and population policy to control or monitor fertility rates (3).

Rapid population growth has been observed in developing countries, including Sub-Saharan African countries, with an estimated population of 1.2 billion by 2025 (9), and of the 175 million under-five children, 45% are chronically undernourished, eclipsing even sub-Saharan Africa. The East Africa region including Ethiopia,has been reported as having the highest prevalence of stunting among children worldwide (4, 10, 11). The prevalence of stunting was 38% based on the 2016 Ethiopian Demographic and Health Survey (EDHS) data and 37% in Ethiopia based on the Ethiopian Mini Demographic and Health Survey (EMDHS) (12, 13). To effectively distribute family education and maternal and child health services to communities with prevalent high-risk fertility behaviors, it is important to follow certain steps or strategies, including conducting a needs assessment to understand high-risk fertility behaviors and community attitudes, engaging stakeholders for culturally appropriate interventions, developing accessible educational materials using local languages and examples, training community health workers, and establishing mobile clinics for maternal and child health services (14, 15).

Several studies identified that early motherhood, high parity, short birth interval, premature labor, anemia, hypertension, gestational diabetes, obesity, pregnancy-related complications, higher rates of cesarean, unsafe abortions, preterm births, intrauterine growth restriction, stillbirths, amniotic fluid embolism, chromosomal abnormalities, and low-birth-weight newborns were triggering factors for child stunting (5, 6, 16–19).

Moreover, due to increased family planning use, expansion of women’s education, and economic trends, pooled decomposition analysis revealed that high-risk fertility behavior was decreased over decades; high-risk fertility behavior of birth is the main cause of under-five children stunting (5, 20–24).

To reduce high-risk fertility behaviors, particularly considering the cultural context, engage community leaders, elders, and influential figures in discussions about the importance of lowering high-risk fertility behaviors. By involving key community members in the planning and implementation of interventions, we can gain their support and help address cultural barriers, design interventions that are culturally appropriate and respectful of traditional norms and values, work with local stakeholders to ensure that interventions are tailored to the specific needs and beliefs of the community, and provide education and awareness programs that address misconceptions and myths surrounding childbirth and pregnancy (15, 25). Many African continents share the same socio-demographic and cultural characteristics where child stunting remains high. HRFB of birth is more prevalent in low-income countries due to widespread poverty, a lack of basic health services, and early child marriage (5, 11, 16, 26, 27). Moreover, children may have a greater probability of chronic under nutrition if they were born from mothers who are too old or too young, if they are born after a short birth interval, or if they are of high birth order (19, 26–28). Prior studies have attempted to pinpoint several variables influencing HRFB of birth and stunting separately (2, 3, 11, 15, 20, 28–33). Even though the causes of stunting are multidirectional, maternal age at delivery, birth spacing between consecutive, and birth order are rubric risk factors for child stunting (5, 16). Largely high-risk fertility behavior of birth is a crucial global health issue for under-five children; it has, over the years, prompted several studies of inquiry. Some studies have reported behaviors such as too-early or too-late mother’s age at delivery, shorter birth interval, birth order, and a higher number of live births to mothers as high-risk fertility behavior that often led to adverse maternal and child health outcomes.

The study conducted based on the 2019 EMDHS revealed that the prevalence of high-risk fertility behavior among reproductive-age women in Ethiopia was 73.50%, and nearly three-fourths of women in Ethiopia had high-risk fertility behavior, which is a key factor for child health. Primary education and a lack of education significantly raise the risk of HRFB (34). In Ethiopia, the risk of experiencing HRFB is reduced through modern contraceptive methods, awareness of modern contraceptive methods, and years of education. High-risk fertility behavior of birth was a prominent cause of child stunting (28). In Ethiopian communities, traditional norms and cultural values play a significant role in influencing high-risk fertility behavior during birth. In Ethiopian culture, having a large family is often seen as a sign of wealth, status, and blessing, and women who choose to seek medical help may be viewed as weak or unable to handle the challenges of childbirth, leading them to avoid seeking necessary care. As a result, there is a strong societal pressure to have many children, which can lead women to continue to have children even if they are at risk of complications during birth. Many Ethiopians believe that events, including childbirth, are predetermined by fate or destiny. This belief can lead women to accept high-risk pregnancies and births as part of their fate without seeking appropriate medical care or interventions. In many rural areas of Ethiopia, access to healthcare facilities and skilled birth attendants is limited (28, 31, 34).

High-risk fertility behavior is linked with numerous unfavorable child outcomes such as chronic under nutrition, anemia, and child mortality (18, 23, 35). However, most previous studies ignore the potential correlation between the HRFB of birth and stunting that failed to show their association statistically in Ethiopia. The proper health of children can be keenly soared by securing HRFB of birth and stunting status of the child (SSC) jointly, drawing back on most previous researchers.

To date, there is a lack of comprehensive research that has examined the association between HRFB of birth and child stunting under five, and no studies have investigated whether poor child health outcomes are influenced by high-risk fertility behavior during birth (HRFB) in bivariate analysis, based on our findings from multilevel analysis view of point. Additionally, many previous studies have primarily utilized enumeration areas as the second level in their multilevel models, while our study employs a new approach using zones instead of enumeration areas. This change is very important as enumeration areas may not be familiar or easily understood by stakeholders, policymakers, or the general public. Based on this, we propose a new model based on both the marginal and conditional probabilities of the correlated binary events such that the joint function can be specified fully by unifying the marginal and conditional probabilities using multilevel analysis. Moreover, the demographic features of Ethiopia are unique because the population’s growth rate and birth and death rates are relatively higher than in other geographical regions; understanding high-risk fertility behavior of birth and sub-regional differentials in the context of reproductive choices would help improve the health and wellbeing of both women and their children in Ethiopia. Therefore, to handle this entire drawback, we stand to examine the nature of the association between HRFB during birth and child health outcomes using bivariate multilevel analysis with data from the Ethiopian Mini Demographic and Health Survey of 2019 data.

## Materials and methods

### Study area, data sources, and study population

This study was conducted in Ethiopia. Ethiopia is the second most populous nation in Africa, with over 110 million people as of 2022 (28, 36). Ethiopia has nine regional states and two administrative cities. Each region is divided into zones, zones into administrative units called Woredas, and Woredas into kebeles (28, 36). The data used for this study were secondary and were obtained from the 2019 Ethiopian Mini Demographic and Health Survey (EMDHS). Approximately 4,969 under-five children who had complete information about HRFB and stunting were considered as the study’s population. In the years 2000, 2005, 2011, and 2016, four full-scale DHS surveys were conducted. The first EMDHS took place in 2014, and the second took place in 2019. EMDHS is usually conducted between 2 and 3 years after the full EDHS is conducted. The 2019 EMDHS was conducted by the Ethiopian Public Health Institute in partnership with the Central Statistical Agency (CSA) and the Federal Ministry of Health between 21 March and 28 June 2019. A national survey called the EMDHS collects data on men, women, and children. A two-stage sampling process was used to collect data from 9 regional states and 2 city administrations. With probability proportional to enumeration area (EA) size based on the 2019 primary healthcare frame and independent selection in each sample stratum, a total of 305 EAs (93 in urban and 212 in rural) were chosen in the first stage. Lists of houses were used as a sampling frame for the selection of households in the second stage so that more specific information could be found elsewhere. Data collection procedures are based on 305 selected EAs with probability proportional to EAs size by independent selection in each sampling stratum of regions in the first stage. In the second stage of selection, a fixed number of 30 households per cluster were selected with an equal probability systematic sampling from the newly created household listing.

### Inclusion and exclusion criteria

In this study, inclusion and exclusion criteria for the sample were used, and we included ever-married reproductive-aged women (15–49 years) and ever-married women who had at least one child born in the 5 years preceding the survey. In addition, we include only children under 5 years who have complete information about stunting and HRFB of birth. Focusing on children under five in a study allows researchers to gain insights into early childhood development, health outcomes, and interventions that can have a significant impact on children’s lives. Children under five are a particularly vulnerable population due to their physical and cognitive development. Studying this age group allows researchers to understand the unique health and development needs of young children. In this study sample, we excluded women who never married and women who had no births.

### Operational definition high-risk fertility behavior of birth

The presence of any one of the following conditions was termed as a single high-risk fertility behavior of birth:

- Birth comes from mothers whose age is less than 18 years at the time of birth,
- Birth comes from mothers whose age is over 34 years at the time of childbirth,
- Latest child born less than 24 months after the previous birth and
- Latest child’s birth order is 3 or higher (28, 36).

However, the birth experiences more than one of these problems at a time, subjected to multiple HRFB of birth. As a whole, if any one of the four criteria mentioned above were met, birth is considered to have experienced high-risk fertility behavior. As a result, a dummy variable was constructed to have a value of 1 if a birth has experienced a high-risk fertility behavior and 0 otherwise.

### Operational definition of stunting

Children whose height-for-age Z-score is below minus two standard deviations (−2 SD) from the median of the reference population are considered short for their age (stunted) or chronically undernourished in our second outcome variable (3, 10, 16, 37).

### Outcome and explanatory variables

The outcome variable for this study was developed based on high-risk fertility behavior of birth and stunting status of under-five children. High-risk fertility behavior of birth and stunting were developed using the definition of the EMDHS (28). This study considered three variables to define the high-risk-fertility behavior of birth: maternal age at the time of delivery, high parity, and short birth interval, which are regarded as bio-demographic factors. When children whose height- for-age Z-score is below two standard deviation(−2SD) from the median of the reference population is know as stunting. The selected socio-demographic and community-level variables included in the analysis are age of under-five children in months, sex of the child, child twin, current age of mothers, current marital status, education level of mother, household wealth index, access to mass media, sex of household head, religion, region of mother, place of residence, contraceptive use, antenatal care follow-up, place delivery, last birth a cesarean section, postnatal care and during pregnancy counseling of health worker, community education level, community wealth index community-level media exposure, and community-level antennal visit were included as community level variables in the study (22, 28, 31, 38).

### Statistical analysis

#### Multilevel bivariate logistic regression

Multilevel bivariate binary logit models are one of the families of multivariate logit models and are used to model the relationships between two correlated binary responses with one or more covariates by considering covariates measured at various levels of a hierarchical structure that affect the outcome variable and how the interactions among covariates measured at different levels affect the outcome variable (39–43).

The Multilevel binary two-level model is given as follows:


(1) $$\mathrm{logit}\left(\pi_{ij}\right)=\log\left(\frac{\pi_{ij}}{1-\pi_{ij}}\right)=\beta_{0j}+\beta_{kj}X_{kij}+\gamma_{0q}Z_{qj\ldots }$$

Where $\beta_{0j}$ is the average intercept, determined as: $\beta_{0j}=\gamma_{00}+U_{0j}$ and $\beta_{kj}=\gamma_{k0}+U_{kj\text{.}}$

Now, the above two-level model (Equation 1) can be rewritten as more generally containing all levels of variation:


(2) $$\begin{array}{l} \mathrm{logit}\left(\pi_{ij}\right)=\log\left(\frac{\pi_{ij}}{1-\pi_{ij}}\right)=\gamma_{00}+\gamma_{k0}X_{kij}+\gamma_{0q}Z_{qj}\\ +U_{0j}+U_{kj}\ldots \end{array}$$

Where $\gamma_{00}$ is the average regression intercept, $X_{kij}$ represents the 𝑘 level-one covariates, and $Z_{qj}$ represents the 𝑞 level-two covariates, whereas, $\gamma_{k0}$ and $\gamma_{0q}$ are the corresponding regression coefficients for level-one and level-two explanatory variables, $U_{0j}$ and $U_{kj}$ are the random effects of the model parameters at level two. Akaike’s information criterion (AIC) and the Bayesian information criterion (BIC) determine the best multilevel model in this study. It is expected that the vector of random effects $U_{0j},U_{1j},U_{2j}\ldots ,U_{kj}$ follows a normal distribution with mean zero and variance $\sigma_{u0}^{2}$ .

The intra-class correlation (ICC) is a measure of the similarity between two people from the same neighborhood and was taken as a value between 0 and 1 inclusive. If there were no variation between the area effects, then all of the $U_{0j}$ would be zero and $\sigma_{u0}^{2}$ would be zero, meaning that ICC = 0. When the ICC is zero, performing a multilevel analysis is not suitable.

ICC was used to determine the proportion of cluster-level variance compared to the total variance that expresses the proportion of the total variance at the cluster level. To observe random effects, two commonly used measures are the intra-class correlation (ICC) and the proportional change in variance (PCV). The intercept-only model decomposes the variance into two independent components. We know that $Y{\_}_{ij}=\gamma {\_}_{00}+U{\_}_{0j}+\epsilon \_ij$ from this $\mathrm{var}\left(Y_{ij}\right)=\mathrm{var}\left(U_{0j}\right)+\mathrm{var}\left(\epsilon_{ij}\right)=\sigma_{u0}^{2}+\sigma_{e}^{2}$ . Covariance between two individuals ( $i\neq j^{\prime }$ ) in the same group: $cov\left(Y_{ij},Y_{i’j}\right)=var\left(U_{0j}\right)=\sigma_{u0}^{2}\text{.}$

Using this model, we can define the ICC by the equation:


(3) $$ICC=\rho \left(Y_{ij},Y_{i’j}\right)=\frac{cov\left(Y_{ij},Y_{i’j}\right)}{var\left(U_{0j}\right)+var\left(\epsilon_{ij}\right)}=\frac{\sigma_{u0}^{2}}{\sigma_{u0}^{2}+\sigma_{e}^{2}}$$

Where, $\sigma_{e}^{2}$ is the variance of individual-level units and $\sigma_{u0}^{2}$ is the variance of the higher-level residual errors. In the multilevel logit modβel, the estimated level-1 variance is the standard logistic distribution π23 which is considered a level 1 residual variance σe2,i.e.,σe2=π23≈3.29 .

Therefore, the above Equation (3) can be rewritten as:


(4) $$ICC=\frac{\sigma_{u0}^{2}}{\sigma_{u0}^{2}+\frac{\pi^{2}}{3}}$$

In the absence of a multilevel structure, a single-level individual analysis is appropriate. Then, there is a proportional change in variance (PCV) or coefficient determination ( $R^{2}$ ), which shows how much the proportion of variance is explained by level 1 and level 2 variables. The proportion of variance explained at the lower level (individual level) is determined as:


(5) $$PCV=R_{1}^{2}=\frac{\sigma_{e|b}^{2}-\sigma_{e|m}^{2}}{\sigma_{e|b}^{2}}$$

Where $\sigma_{e|b}^{2}$ is the lowest level residual variance for the baseline model, which is the intercept-only model, and $\sigma_{e|m}^{2}$ is the lowest residual variance for the comparison model, while the proportion of variance explained at the highest level (community level) is determined as:


(6) $$R_{2}^{2}=\frac{\sigma_{u0|b}^{2}-\sigma_{u0|m}^{2}}{\sigma_{u0|b}^{2}}$$

Where, $\sigma_{u0|b}^{2}$ is the highest-level residual variance for the baseline model and $\sigma_{u0|m}^{2}$ is the highest-level residual variance for the comparison model.

Let $Y_{1}$ and $Y_{2}$ be stunting status and HRFB of birth of this study, then the two bivariate binary responses denoted as a vector of Y = ${\left(\gamma_{11}\;\gamma_{01}\;\gamma_{10}\;\gamma_{00}\right]}^{T}$ . The elements of y have the probabilities of $\pi_{11}$ , $\pi_{10}$ , $\pi_{01}\text{,}$ and $\pi_{00}$ , respectively, which are presented in Table 1.

**Table 1:** The joint probability of the response variables.

$Y_{2}$ = HRFB of birth				
$Y_{1}$ = Stunting status		1	0	Total
	1	$\pi_{11}$ = p( $y_{1\;=}1y_{2}=1)$	$\pi_{10}$ = p( $y_{1\;=}1y_{2}=0)$	$\pi_{1}$
	0	$\pi_{01}$ = p( $y_{1=}0y_{2}=1)$	$\pi_{00}=p$ ( $y_{1\;=}0y_{2}=0)$	1- $\pi_{1}$
Total		$\pi_{2}$	1- $\pi_{2}$	1

Moreover, the bivariate multilevel Logistic regression model can be written as follows:


(7) $$\begin{array}{l} g_{1}\;\left(x\right)=\mathrm{logit}\;\left(\pi_{1}\;\left(x\right)\right)=\beta_{1}x^{T},g_{2}\;\left(x\right)=\mathrm{logit}\;\left(\pi_{2}\;\left(x\right)\right)=\\ \beta_{2}x^{T}g_{3}\left(x\right)=\mathrm{lnln\psi}\left(x\right)=\beta_{3}x^{T} \end{array}$$

The odds ratio (OR), which is used to describe the dependence of the two outcomes, HRFB of birth and child stunting status, using the basic link function of logit, is stated as


(8) $$\Psi =\frac{P\;r\left(Y2=1\;|\;Y1=1\right)\;}{P\;r\left(Y2=1\;|\;Y1=0\right)}=\frac{\pi_{00}}{\pi_{01}}\frac{\pi_{11}}{\pi_{10}},\;\mathrm{where}\;\Psi \geq 0$$

Where 𝑥 = [1 $x_{1}x_{2}$ ⋯ $x_{k}{]}^{T}\text{,}$ is a vector of the covariate, ${\beta_{1}}^{T}$ = [ $\beta_{01}\;\beta_{11\;}\beta_{12}$ ⋯ $\beta_{1K}$ ], ${\beta_{2}}^{T}$ = [ $\beta_{02}\;\beta_{12\;}\beta_{22}$ ⋯ $\beta_{2K}$ ], and ${\beta_{3}}^{T}$ = [ $\beta_{03}\;\beta_{13\;}\beta_{23}$ ⋯ $\beta_{K3}$ ] are the parameters, $\pi_{1}\left(x\right)$ is the marginal probability function of $Y_{1}$ , $\pi_{2}\left(x\right)$ is the marginal probability function of $Y_{2}$ , and Ψ (𝑥) is the odds ratio that shows the association between $Y_{1}$ and $Y_{2}$ .

Then, the joint probability function is,

$p\;\left(y_{1},y_{2}\right)=\;\overset{1}{\underset{j=0}{\prod }}\overset{1}{\underset{k=0}{\prod }}{\left[P\left(Y2=k|Y_{1}=j\right)\right]}^{y_{jk}}\text{,}$ where; $\beta_{1}$ = ${\left(\beta_{10},\;\beta_{11,\;}\beta_{12},.\;.\;.,\beta_{1p}\right)}^{T}$


(9) $$\begin{array}{l} \mathrm{OR}=\pi_{j\left(X\right)}=\frac{e^{\beta j0+\beta j1X1+\beta j2X2+\beta j3X3+\ldots .+\mathrm{\beta jpXp}}}{1+e^{\beta jo+\beta j1X1+\beta j2X2+\beta j3X3+\ldots +\mathrm{\beta jpXP}}}=\\ \frac{e^{X\beta j}}{1+e^{X\beta j}}\;j=\;1,\;2,\ldots \end{array}$$

The null hypothesis $H_{0}$ : $\beta_{11}$ = $\beta_{10}$ can be tested for independence in the presence of covariates between the two binary outcome variables $Y_{1}$ and $Y_{2}$ (40, 41).

To obtain the parameters estimator of the model, the maximum likelihood method was applied using the Newton–Raphson algorithm iterative procedure. The maximum likelihood estimator can be obtained by determining the first partial derivatives of the log-likelihood function and then equating them to zero (41, 44).

## Results

In this study, a total weighted sample of 4,969 under-five children was included. According to the findings of this study, 36.8% of children under the age of 5 have stunted growth and 60% of births fall into avoidable risk of HRFB for birth. Approximately 1,177(24%) children were stunted due to high-risk fertility behavior.

The study results revealed that the majority of participants (3,676) were found in the age range of 19–34, and this age group had the highest prevalence of HRFB, which accounts for approximately 50.4%. Among 4,969 under-five children who were included in this study, approximately one-third (1805) were Muslims, with the highest percentage of HRFB contributing approximately 40.2%. However, a high prevalence of child stunting was observed among those who were orthodox (35.7%). In the same result, around 75% of under-five children were from rural areas, with an 80.8% prevalence of HRFB and 82.5% prevalence of stunted children. Furthermore, approximately 54% of mothers (2665) had never attended school, with 68.1% of them experiencing HRFB and 60.7% of their children being stunted. Moreover, in terms of the origin of the place, the majority of under-five children were from the Oromia region, where 42.9% of HRFB and 38.7% of stunting cases were reported. There were 301 (6%) mothers who gave birth before the age of 18 years, contributing to 10% of HRFB cases, and 136 (7.4%) of their births were stunted. On the other hand, 594 (12%) of the mothers gave birth above the age of 34 years, contributing to 19.9% of HRFB cases and 9.1% of stunting cases.

Among the births, 2,295 came from high birth order; of them, approximately 76.8% had HRFB cases and 48.9% of stunting cases. Short birth intervals resulted in 925 births, with 30.1% considered as HRFB and 21% of them being stunted. In the same result, the majority of mothers in the study had breastfed for their children, 3,531 (71%), and 69.4% of their subsequent children experienced HRFB, with 69% being stunted. The study also indicated that approximately 1,698 (34%) had access to media; of them, approximately 27.6% experienced HRFB, while 27% had stunted children. Moreover, the majority of mothers, 4,716 (95%), did not undergo cesarean section deliveries, resulting in a prevalence of 95.6% for HRFB and 95.4% for stunting. Two-thirds of the respondents, 3,030 (61%), did not consult a health worker during pregnancy, and among them, 65.5% of their births were categorized as HRFB, with 63.6% of their children being stunted. Similarly, the results demonstrated that approximately 2,885 (58%) of the respondents did not use contraceptive methods, leaving 65.3% exposed to the problem of HRFB. Moreover, the majority of respondents, 4,462 (90%), did not undergo postnatal care checkups, contributing to 90.6% of HRFB cases (Table 2).

**Table 2:** Distribution and association of covariate with HRFB of birth and SSC.

Independent variables with categories	Frequency (%)	HRFB of birth		SSC	
		Yes (%)	$\chi^{2}-$ *p*-value		Yes $\chi^{2}-$ *p*-value
Age of mother (in year)					
15–18 19–34 35–49	147(3)	141(4.7)	0.0001	55(3)	0.2838
	3,676(74)	1778(50.4)		1,376(75.2)	
	1,146(23)	1,072(45.8)		399(21.8)	
Religion of mother					
Orthodox Catholic Protestant Muslim Others	1705(34)	882(29.5)	0.0001	686(35.4)	< 0.0001
	17(1)	12(0.4)		6(0.3)	
	1,362(27)	832(27.8)		470(27.7)	
	1805(36)	1,203(40.2)		653(35.7)	
	80(2)	62(2.)		15(0.9)	
Place of residence					
Urban Rural	1,249(25)	573(19.2)	0.0001	320(17.5)	< 0.0001
	3,720(75)	2,418(80.8)		1,510(82.5)	
Ever attended school					
No Yes	2,665(54)	2036(68.1)	0.0001	1,111(60.7)	< 0.0001
	2,304(46)	955(31.9)		719(39.3)	
Mother educational level					
No education Primary Secondary Higher	2,665(53)	2036(52.8)	0.0000	1,111(62.4)	0.0001
	1757(35.6)	814(26.4)		618(22.5)	
	366(7.4)	103(20.1)		70(18.4)	
	181(3.6)	37(0.7)		31(0.6)	
Region					
Tigray Afar Amhara Oromia Somali Benishangul SNNPR Gambela Harari Addis Ababa Dire Dewa	351(7)	172(5.8)	0.0001	172(9.4)	<0.0001
	74(2)	50(1.7)		32(1.7)	
	957(19)	500(16.7)		391(21.4)	
	1978(36)	1,284(42.9)		708(38.7)	
	343(7)	265(8.9)		105(5.7)	
	60(2)	36(1.2)		24(1.3)	
	1,005(19)	614(20.5)		364(19.9)	
	22(1)	11(0.4)		4(0.2)	
	14(1)	8(0.3)		5(0.3)	
	138(3)	37(1.2)		19(1)	
	25(1)	13(0.4)		7(0.4)	
Wealth index					
Poorest Poorer Middle Richer Richest	1,140(23%)	824(27.5)	0.0001	486(26.6)	< 0.0001
	1,098(22%)	728(24.3)		426(23.3)	
	931(18)	617(20.6)		392(21.4)	
	80(17)	511(17.1)		318(17.4)	
	917(18)	311(10.4)		207(11.3)	
Age of under five children in months					
0–5 6–11 12–23 24–35 36–47 48–59	529(11)	316(10.6)	0.0001	90(4.9)	< 0.0001
	473(10)	241(8.1)		131(7.2)	
	1,002(20)	574(19.2)		328(17.9)	
	979(20)	626(20.9)		445(24.3)	
	1,041(21)	641(21.4)		427(23.3)	
	941(18)	593(19.8)		409(22.3)	
Sex of child					
Male Female	2,529(51)	1,494(49.9)	0.108	1,012(55.3)	< 0.0001
	2,440(49)	1,497(50.1)		818(44.7)	
Child twin					
Yes No	106(2)	76(2.5)	0.0109	60(3.3)	< 0.0001
	4,864(98)	2,915(97.5)		1770(96.7)	
Birth from women age below 18					
Yes No	301(6)	301(10.1)	0.0001	136(7.4)	0.0018
	4,668(94)	2,690(89.9)		1,694(92.1)	
Birth from mother age below 34					
Yes No	594(12)	594(19.9)	0.0001	166(9.1)	< 0.0001
	4,375(88)	2,397(80.1)		1,665(90.1)	
Birth order of child					
High Low	2,295(46)	2,295(76.8)		894(48.9)	0.0043
	2,674(54)	695(23.2)		937(51.1)	
Birth interval of child					
High Low	925(19)	925(30.1)	0.0001	398(21.7)	< 0.0001
	4,044(81)	2066(81)		1,432(78.3)	
	2,510(51)	704(23.5)		863(47.3)	
No of children under five in the house					
0 1 2 3 4 and above	18(0.4)	0(0.0)	0.0001	7(0.4)	< 0.0001
	1944(39)	948(31.7)		652(13.6)	
	2,309(46.6)	1,456(48.7)		893(48.8)	
	597(12)	499(16.7)		250(13.7)	
	100(2)	87(2.9)		27(1.5)	
Number of household members					
Less than five More than five	2,264(46)	727(24.3)	0.0001	794(43.4)	0.0176
	2,705(54)	2,263(75.7)		1,036(56.6)	
Currently pregnant status					
Yes No	524(11)	336(11.2)	0.2575	208(11.4)	0.2752
	4,445(89)	2,655(88.8)		1,622(88.6)	
Currently breastfeeding status					
Yes No	3,531(71)	2,075(69.4)	0.6014	1,263(69)	0.7166
	1,439(29)	916(30.6)		567(31)	
Marital status					
Yes No	4,685(94)	2,830(94.6)	0.2897	1738(95)	0.3020
	285(6)	161(5.4)		91(5)	
Sex of house hold head					
Male Female	4,329(87)	2,613(87.4)	0.5264	1,641(89.7)	< 0.0001
	641(13)	378(12.6)		189(10.3)	
Media exposures					
Yes No	1,698(34)	825(27.6)	<0.0001	494(27)	< 0.0001
	3,272(66)	2,165(72.4)		1,336(73)	
Delivery by caesarean section					
Yes No	253(5)	133(4.4)	0.0112	84(4.6)	0.2033
	4,716(95)	2,858(95.6)		1746(95.4)	
During pregnancy counseling health worker					
Yes No	1939(39)	1,033(34.5)	<0.0001	666(36.4)	0.0039
	3,030(61)	1958(65.5)		1,164(63.6)	
Current contraceptive use					
Yes No	2085(42)	1,039(34.7)	<0.0001	739(40.4)	0.0786
	2,885(58)	1952(65.3)		1,092(59.6)	
Postnatal check within 2 months					
Yes No	508(10)	281(9.4)	0.0174	181(9.9)	0.5265
	4,462(90)	2,710(90.6)		1,649(90.1)	
Number of anti-natal care (ANC) visit					
No visit 1–3 visit 4 and above	896(18)	682(22.8)	<0.0001	299(16.3)	0.0004
	2,500(50)	1,534(51.3)		987(54.0)	
	1,574(32)	775(25.9)		543(29.7)	
Place of delivery					
Health facility Home	2,432(48)	1,164(38.9)	<0.0001	826(45.1)	<0.0001
	2,538(52)	1827(61.1)		1,004(45.9)	

Approximately 61.9% of mothers in the community were unable to read or write. Among 61.9% of mothers who did not read or write, approximately 35.4% of their births had a high risk of fetal birth and 1,222 (66.8%) of the children were stunted. Approximately 45.8% of the community mothers came from poor families; of them, half of the births were classified as HRFB of birth cases and 869 (47.5%) of the children were stunted. Based on the same findings, approximately 46.2% of the mothers did not have access to media, and among them, 1,452 (48.5%) of the births were considered to have HRFB of birth, with 869 (49.0%) of their children being stunted. Similarly, approximately 56.5% of community mothers received inadequate antenatal care during pregnancy. Among these mothers, 1917 (64.1%) of their children were at risk of high-risk fetal birth, and more than half (57.7%) suffered from stunting (Table 3).

**Table 3:** Distribution and association of community-level covariates with HRFB of birth and SSC.

Community level variable	Frequency (%)	HRFB of Birth			SSC
		Yes (%)	$\chi^{2}-$ *P*-value	Yes (%)	$\chi^{2}-$ *P*-value
Mothers education					
Low	3,076(61.9)	1933(64.6)	0.0000	1,222(66.8)	0.0000
High	1893(38.1)	1,058(35.4)		609(33.2)	
Wealth index					
Low	2,274(45.8)	1,479(49.4)	0.0000	869(47.5)	0.0000
High	2,695(54.2)	1,512(50.6)		961(52.5)	
Media exposure					
Low	2,295(46.2)	1,452(48.5)	0.0000	869(49.0)	0.0000
High	2,675(53.8)	1,539(51.5)		934(51.0)	
Antenatal care					
Low	2,806(56.5)	1917(64.1)	0.0000	1,056(57.7)	0.0000
High	2,164(43.5)	1,074(35.9)		774(42.3)	

The findings of this study revealed that the odds ratio between HRFB of birth and stunting was 1.32 [OR = 1.32, 95% CI: (1.17–1.48)], which is different from 1, indicating that there is a statistically significant association between HRFB of birth and stunting. Additionally, the results suggest that 1,177 children (24%) of the sample experienced stunting as a result of inheriting the high-risk fertility behavior (Table 4).

**Table 4:** Joint and marginal probability of HRFB of birth and SSC.

Responses		SSC		Marginal of HRFB	Odds ratio (95% CI)
		No	Yes		
HRFB	No	1,325(0.267)	653(0.128)	1977(0.3981)	1.32(1.17,1.48)
	Yes	1814(0.365)	1,177(0.240)	2,992(0.6019)	
Marginal of SSC		3,139(0.632)	1830(0.368)	4,969(1)	

Measure of association, $\chi^{2}\;=20.52\text{,}$ P:value=0.0001 .

The results of this study indicate that the highest prevalence of both HRFB of birth and stunting was observed among mothers in the age group of 19–34 years, with a total of 758 cases (64.4%). Among different religious groups in Ethiopia, Muslims had the highest prevalence of HRFB of birth and stunting, with 452 cases (38.4%). Similarly, in terms of residential areas, the majority of HRFB of birth and stunting cases were found among mothers residing in rural areas, totaling 999 cases (84.9%). Furthermore, the study revealed that three-fourths of the respondents had not received any formal education, resulting in the highest incidence of HRFB of birth and stunting, with 860 cases (73.1%). The majority of mothers without access to media were also exposed to both HRFB of birth and stunting, with a total of 906 cases (77%). Male household heads were found to contribute significantly to HRFB of birth and stunting, with 1,062 cases (90.2%). Approximately 55.4% of the children were stunted, and 65% were at a high risk of birth if their mothers did not use contraceptives. The study also revealed that mothers from the poorest households were more likely to experience HRFB of birth and stunting, with 358 cases (30%). Additionally, the minimum and maximum prevalence of HRFB of birth and stunting were observed in children aged 0–5 months and 48–59 months, respectively. Out of the total male children in the study, 652 cases (55.4%) were found to be stunted and exposed to HRFB of birth (Table 5).

**Table 5:** Joint distribution of explanatory variables for the different combinations of HRFB of birth and SSC.

Independent variables	Association of HRFB of birth and SSC				$\chi^{2}-$ *P*-value
	No HRFB and Non-SSC (%)	HRFB and Non- SSC (%)	No HRFB and SSC (%)	HRFB and SSC (%)	
Mother’s age in year					
15–18 19–34 35–49	5(0.4)	87(4.8)	1(0.2)	54(4.6)	0.0001
	1,280(96.6)	1,020(56.2)	618(94.6)	758(64.4)	
	40(3)	707(39)	34(5.2)	365(31)	
Religion of mother					
Orthodox Catholic Protestant Muslim Others	543(41)	477(26.3)	280(42.9)	405(34.4)	0.0001
	0(0)	8(0.4)	3(0.5)	3(0.3)	
	369(27.8)	524(28.9)	161(24.7)	309(26.3)	
	401(30.3)	751(41.4)	201(30.8)	452(38.4)	
	12(0.9)	54(3)	8(1.2)	7(0.6)	
Place of residence					
Urban Rural	534(40.3)	395(21.8)	143(21.9)	177(15.1)	0.0001
	792(59.7)	1,419(78.2)	511(78.1)	999(84.9)	
Ever attended school					
No Yes	378(28.5)	1,177(64.9)	251(38.4)	859(73.1)	0.0000
	948(71.5)	637(35.1)	402(61.6)	317(26.9)	
Mother educational level					
No education	378(28.2)	1,177(64.9)	251(38.4)	860(73.1)	0.0000
Primary	617(46.5)	522(28.8)	326(49.9)	292(24.8)	
Secondary	214(16.1)	82(4.5)	49(7.5)	21(1.8)	
Higher	117(8.8)	33(1.8)	27(4.1)	4(0.3)	
Region					
Tigray Afar Amhara Oromia Somali Benishangul SNNPR Gambela Harari Addis Ababa Dire Dewa	107(8.1)	73(4)	73(11.2)	99(8.4)	0.0000
	15(1.1)	27(1.5)	9(1.4)	23(2)	
	297(22.4)	269(14.8)	161(24.6)	230(19.6)	
	474(35.7)	797(44)	221(33.8)	487(41.4)	
	59(4.4)	180(9.9)	20(3.1)	85(7.2)	
	14(1.1)	20(1.1)	9(1.4)	15(1.3)	
	250(18.9)	392(21.6)	141(21.6)	223(19)	
	9(0.7)	9(0.5)	1(0.2)	2(0.2)	
	5(0.4)	4(0.2)	1(0.2)	4(0.3)	
	86(6.5)	33(1.8)	15(2.3)	4(0.3)	
	10(0.8)	9(0.5)	3(0.5)	4(0.3)	
Wealth index of household					0.0000
Poorest Poorer Middle Richer Richest	188(14.2)	466(25.7)	128(19.6)	358(30.4)	
	226(17)	447(24.6)	144(22.1)	282(23.9)	
	203(15.3)	336(18.5)	112(17.2)	281(23.9)	
	243(18.3)	320(17.6)	127(19.4)	191(16.2)	
	466(35.1)	245(13.5)	142(21.7)	66(5.6)	
Number of under five in household					
0 1 2 3 4 and above	11(0.8)	0(0)	7(1.1)	0(0)	0.0000
	699(52.8)	594(32.7)	297(45.6)	355(30.2)	
	544(41.1)	872(48.1)	309(47.4)	584(49.6)	
	59(4.5)	287(15.8)	38(5.8)	212(18)	
	12(0.9)	61(3.4)	1(0.2)	26(2.2)	
Age of under five children in months					
0–5 6–11 12–23 24–35 36–47 48–59	181(13.6)	258(14.2)	32(4.9)	58(4.9)	0.0001
	183(13.8)	160(8.8)	51(7.8)	80(6.8)	
	293(22.1)	382(21.1)	136(20.8)	192(16.3)	
	196(14.8)	339(18.7)	158(24.2)	287(24.4)	
	252(19)	363(20)	149(22.8)	278(23.6)	
	222(16.7)	312(17.2)	128(19.6)	281(23.9)	
Sex of child					
Male Female	676(51)	841(46.4)	359(55)	652(55.4)	0.0000
	650(49)	973(53.6)	294(45)	525(44.6)	
Child is twin					
No Yes	1,317(99.4)	1776(97.9)	632(96.8)	1,139(96.8)	0.0000
	8(0.6)	38(2.1)	21(3.2)	38(3.2)	
Birth from women age below 18					
No Yes	1,325(100)	1,649(90.9)	653(100)	1,041(88.4)	0.0000
	0(0)	165(9.1)	0(0)	136(11.6)	
Birth from women age above 34					
No Yes	1,325(100)	1,385(76.4)	653(100)	1,011(85.9)	0.0001
	0(0)	429(23.6)	0(0)	166(14.1)	
Birth order of child					
Low High	1,325(100)	412(22.7)	653(100)	283(24)	0.0001
	0(0)	1,402(77.3)	0(0)	894(76)	
Birth interval of child					
Low High	1,325(100)	1,287(70.9)	653(100)	779(66.2)	0.0001
	0(0)	527(29.1)	0(0)	398(33.8)	
Number of household members					
≤5 ≥6	1,033(78)	437(24.1)	503(77)	290(24.6)	0.0000
	292(22)	1,377(75.9)	150(23)	887(75.4)	
Sex of house hold head					
Male Female	1,137(85.8)	1,551(85.5)	539(88.7)	1,062(90.2)	0.0000
	188(14.2)	263(14.5)	74(11.3)	115(9.8)	
Pregnancy counseling					
No	705(53.2)	1,162(64.1)	368(56.4)	797(67.6)	0.0000
Yes	621(46.8)	652(35.9)	285(43.6)	381(32.4)	
Media exposures					
No Yes	676(51)	1,259(69.4)	430(65.8)	906(77)	0.0000
	649(49)	555(30.6)	223(34.2)	271(23)	
Delivery by caesarean section					
No Yes	1,253(94.5)	1717(94.7)	606(92.8)	1,140(96.9)	0.0000
	73(5.5)	97(5.3)	47(7.2)	36(3.1)	
Current contraceptive use					
No Yes	602(45.4)	1,190(65.6)	330(50.5)	762(64.7)	0.0001
	723(54.6)	624(34.4)	324(49.5)	415(35.3)	
Postnatal check within 2 months					
No Yes	1,161(87.6)	1,651(91)	590(90.4)	1,059(90)	0.0000
	164(12.4)	163(9)	63(9.6)	118(10)	
Number ANC visit					
No visit 1–3 visit 4 and above	141(10.6)	456(25.1)	72(11)	227(19.3)	0.0000
	632(47.7)	880(48.5)	334(51.1)	654(55.6)	
	552(41.7)	478(26.4)	247(37.8)	296(25.1)	
Place of delivery					
Home Health facility	439(33.1)	1,094(60.3)	271(41.4)	733(62.3)	0.0000
	886(66.9)	720 (39.7)	383(58.6)	443(37.7)	

Among mothers who were from an uneducated community, a significant proportion of their children, specifically 822 cases (69.8%), were found to be at risk of both high-risk fertility behavior (HRFB) and stunting. When examining the wealth index of the family community, it was observed that approximately half of the children were stunted and at risk of HRFB when the family belonged to the poor category. Similarly, nearly half of the children were exposed to both HRFB and stunting when their families had limited access to media. Additionally, children from communities with low antenatal visit rates had an increased likelihood of being exposed to HRFB and stunting; with 1,056 cases (57.7%) affected simultaneously (Table 6).

**Table 6:** Joint multilevel distribution of explanatory variables for HRFB of birth and SSC.

Community (Zonal) Level variable	Multilevel Association of HRFB of birth and SSC (%)				
	No vs. No	Yes vs. No	No vs. Yes	Yes vs. Yes	$\chi^{2}-$ *P*-value
Mothers education					
Low	743(56.0)	1,112(61.3)	400(61.3)	822(69.8)	0.0000
High	583(44.0)	702(38.7)	253(38.7)	355(30.2)	
Wealth index					
Low	518(39.1)	888(49.0)	277(42.4)	592(50.3)	0.0000
High	808(60.9)	926(51.0)	376(57.6)	585(49.7)	
Media exposure					
Low	539(40.7)	860(47.4)	304(46.5)	896(48.9)	0.0000
High	786(59.3)	954(52.6)	350(53.5)	935(51.5)	
Antenatal care					
Low	580(43.7)	1,170(64.5)	309(47.3)	1,056(57.7)	0.0000
High	746(56.3)	644(35.5)	344(52.7)	774(42.3)	

ICC 0.20; AIC 13585.29; BIC 13589.6; PCV (R^2^) 0.78.

The intra-class correlation coefficient (ICC) for this model was 0.2, indicating that 20% of the variation in high-risk fertility behavior of birth and stunting can be attributed to differences between communities (zonal variation). The remaining 78% of the total variation is accounted for by individual-level factors and community-level factors in the multilevel model (Table 6).

The result of this study was found that if the mother was under 18 years old at the time of birth, there was a 1.57 times higher risk of stunting compared to mothers older than 18 years [adjusted odds ratio (AOR) = 1.57, 95% CI: (1.118, 2.208)]. Conversely, mothers aged over 34 years at the time of birth had a 0.56 lower risk of stunting compared to those under 34 years old [AOR = 0.56, 95% CI: (0.457, 0.685)]. Moreover, children from families with a birth order greater than 3 and a birth interval of less than 24 months had a 1.73 and 1.30 times higher risk of stunting, respectively, compared to those with a birth order less than 3 and a birth interval greater than 24 months [AOR = 1.73, 95% CI: (1.036, 1.811) and AOR = 1.30, 95% CI: (1.078, 1.556)]. Additionally, if the birth was from a mother with a single HRFB of birth problem, there was a 1.63 times higher likelihood of stunting compared to the non-risk group [AOR = 1.63, 95% CI: (1.463, 2.865)]. Similarly, if the birth was from a mother with multiple high-risk fertility behaviors, the occurrence of stunting was 1.64 times higher compared to the non-risk group [AOR = 1.64, 95% CI: (1.429, 2.579)]. Overall, if the birth was from a mother with any HRFB of birth problem, the birth had a 1.44 times higher risk of stunting compared to births without HRFB problems [AOR = 1.44, 95% CI: (1.017, 1.974)]. Based on these results, the most significant factors associated with the stunting status of children were births from mothers below 18 years old and high parity (Table 7).

**Table 7:** Joint prevalence, crude, and adjusted odds ratios between HRFB of birth and SSC.

HRFB of birth (%)		SSC (%)		COR(95% CI)	AOR (95% CI)
		Yes No			
Age less than 18 years					
Yes		136(7.4)	165(5.3)	1.45(1.147 1.834)	1.57(1.118 2.208)*
No (Ref.)		1,694(92.6)	2,974(94.7)		
Age above 34 years					
Yes		166(9.1)	429(13.7)	0.63(0.521 0.76)	0.56(0.457 0.685)**
No (Ref.)		1,665(90.9)	3,140(86.3)		
Preceding birth interval less than 24 months					
Yes		398(21.7)	527(16.8)	1.38(1.192 1.594)	1.30(1.078 1.556)*
No (Ref.)		1,432(78.3)	2,613(83.2)		
Birth order 4 and above					
Yes		894(48.9)	1,402(44.6)	1.58(1.054 1.328)	1.73(1.036 1.811)*
No(Ref.)		936(51.1)	1738(55.4)		
Type of HRFB of birth					
Single	Yes	787(43.0)	1,168(37.2)	1.37(1.201 2.558)	1.63(1.463, 2.865)*
Multiple	Yes	390(21.3)	647(20.6)	1.24(1.046 1.432)	1.64(1.429, 2.579)*
No birth	No (Ref.)	653(35.7)	1,325(42.2)		
Any HRFB of birth					
Yes		1,177(64.3)	1,325(42.2)	1.32(1.169 1.483)	1.44(1.017, 1.974)*
No(Ref.)		653(35.7)	1814(57.8)		

* = significant at 𝑎 = 0.05, ** = significant at 𝑎 = 0.01 and *** = significant at 𝑎 = 0.001.

Furthermore, the findings of a multilevel bivariate binary model show that male children had 1.36 times higher odds of stunting compared to female children [AOR = 1.36, 95% CI: (1.19, 1.55)], and estimated odds of HRFB of birth were 0.83 times lower for male children compared to female children. Children from Orthodox, Muslim, Catholic, and Protestant families had an increased likelihood of stunting, particularly for the Muslim religion. Children born into households with a middle wealth index had a 42% higher likelihood of HRFB of birth than those from the poorest wealth index category. The estimated odds of stunting were 2.66 times higher in the Tigray region than women in the capital city, Addis Ababa [AOR = 2.66 95% CI: (1.30, 5.47)]. Mothers with primary, secondary, and higher education had a 0.71, 0.63, and 0.46 odds-time steeply decrease in the estimated odds of HRFB of birth compared to uneducated mothers [AOR = 0.71, 95% CI: (0.22, 0.85)], [AOR = 0.63, 95% CI: (0.40, 0.99)], and [AOR = 0.46, 95% CI: (0.28, 0.74)] respectively, and mothers with primary, secondary, and higher education had a 0.82, 0.49, and 0.52 odds-time reduced in the estimated odds of HRFB of birth compared to uneducated mothers [AOR = 0.82, 95% CI: (0.71, 0.95)], [AOR = 0.49, 95% CI: (0.35, 0.67)], and [AOR = 0.54, 95% CI: (0.36, 0.85)] respectively.

Furthermore, the estimated odds of stunting were 2.67 times higher among twin children than non-twin children [AOR = 2.67; 95% CI: (1.72, 4.14)]. Health facility deliveries were associated with 0.71 times lower odds of HRFB of birth compared to home deliveries [AOR = 0.71, 95% CI: (0.59, 0.84)], which reflects that those children born from complete antenatal visits had lower odds of HRFB of birth but increased risk of stunting for four or more visits (Table 8).

**Table 8:** Parameter estimates of the multilevel bivariate modeling of HRFB of birth and SSC.

Variables	HRFB of birth		SSC	
	Estimate (SE)	OR (95% CI)	Estimate (SE)	OR (95% CI)
Intercept	−2.81(0.73)	0.006(0.014, 0.26) ***	−2.81(0.73)	0.005(0.014, 0.26)***
Age of under five children in months				
0-5(ref)		1		1
6–11	−4.23(1.98)	0.021(0.0003, 0.71) *	0.70(0.17)	2.02(1.46, 2.77) ***
12–23	−3.91(1.97)	0.020(0.0004, 0.96) *	1.10(0.14)	3.00(2.24, 3.90) ***
24–35	−3.64(1.97)	0.027(0.0006, 1.27)	1.65(0.14)	5.21(3.94, 6.90) ***
36–47	−3.71(1.97)	1.57(0.0005, 1.17)	1.51(0.15)	4.53(3.40, 5.99) ***
48–59	−3.79(1.97)	0.019(0.00047, 1.09)	1.53(0.15)	4.62(3.42, 6.17) ***
House hold wealth index				
Poorest(ref)		1		1
Poorer	−0.01(0.12)	0.99(0.78, 1.26)	−0.32(0.10)	0.73(0.60, 0.89) **
Middle	0.35(0.13)	1.42(1.10, 1.84) **	−0.06(0.11)	0.94(0.76, 1.70)
Rich	0.075(0.15)	1.08(0.81, 1.43)	−0.18(0.12)	0.84(0.66, 1.06)
Richest	−0.39(0.9)	0.68(0.43, 0.99) *	−0.52(0.17)	0.59(0.43, 0.83) **
Region				
Addis Ababa(ref)		1		1
Afar	0.42(0.5)	1.52(0.56, 4.14)	0.33(0.45)	1.39(0.56, 3.40)
Amhara	0.53(0.38)	1.70(0.79, 3.61)	0.44(0.35)	1.55(0.78, 3.12)
Benishangul	0.81(0.55)	2.25(0.75, 6.69)	0.55(0.47)	1.73(0.69, 4.44)
Dire Dewa	0.74(0.7)	1.90(0.52, 8.56)	0.18(0.65)	1.20(0.33, 4.33)
Gambela	0.84(0.70)	2.32(0.56, 9.55)	−0.12(0.72)	0.89(0.21, 3.73)
Harari	0.83(0.81)	2.25(0.43, 12.15)	0.63(0.73)	1.88(0.43, 8.12)
Oromia	0.7(0.36)	2.02(0.98, 4.14)	0.17(0.33)	1.18(0.61, 2.32)
SNNPR	0.46(0.37)	1.58(0.74, 3.34)	0.28(0.3)	1.32(0.66, 2.63)
Somalia	0.56(0.44)	1.75(0.73, 4.19)	−0.33(0.39)	0.72(0.33, 1.58)
Tigray	0.74(0.4)	2.09(0.95, 4.67)	0.98(0.36)	2.66(1.30, 5.47) **
Mother’s educational level				
No education(ref)		1		1
Primary	−0.34(0.15)	0.71(0.22, 0.85)***	−0.21(0.08)	0.82(0.71, 0.95)***
Secondary	−0.46(0.23)	0.63(0.40, 0.99) *	−0.72(0.16)	0.49(0.35, 0.67)***
Higher	−0.79(0.25)	0.46(0.28, 0.74)**	−0.62(0.23)	0.54(0.36, 0.85) **
Child is twin				
No(ref)		1		1
Yes	0.64(0.28)	1.90(0.99, 3.32)	0.98(0.23)	2.67(1.72, 4.14)***
Current age of the mother in years				
19-34(ref)		1		1
15–18	4.09(0.43)	59.7(25.50, 139.99)***	0.34(0.19)	1.41(0.96, 2.08) *
35–49	2.96(0.14)	19.29(14.59, 25.61)***	−0.32(0.08)	0.73(0.62, 0.86)***
Sex of child				
Female(ref)		1		1
Male	−0.18(0.08)	0.83(0.72, 0.97) *	0.31(0.63)	1.36(1.19, 1.55)***
Religion of respondents				
Other(ref)		1		1
Orthodox	−0.1(0.35)	0.90(0.45, 1.79)	1.13(0.30)	3.10(1.69, 5.58)**
Muslim	0.31(0.34)	1.36(0.69, 2.68)	1.63(0.63)	5.10(1.47, 17.52)*
Catholic	0.78(0.76)	2.18(0.49, 9.66)	1.14(0.31)	3.13(1.71, 5.76)**
Protestant	0.27(0.33)	1.31(0.68, 2.51)	1.25(0.32)	3.49(1.87, 6.52)*
Number ANC visit				
No Visit(ref)		1		1
1–3 Visit	−0.76(0.13)	0.47(0.36, 0.60)***	0.034(0.11)	1.03(0.84, 1.27)
4 and above	−0.61(0.14)	0.54 (0.41, 0.73)***	0.34(0.12)	1.41(1.10, 1.79)**
Place of residence				
Urban(ref)		1		1
Rural	0.12(0.13)	1.13(0.89, 1.47)	0.35(0.12)	1.42(0.99, 1.79)
During pregnancy: Counseling health worker				
No(ref)		1		1
Yes	0.73(0.11)	2.07(0.99, 2.57)	0.01(0.09)	1.01(0.85, 1.21)
Sex of household head				
Female(ref)		1		1
Male	−0.04(0.18)	0.96(0.68, 1.38)	0.23(0.15)	1.26(0.94, 1.68)
Place of delivery				
Home(ref)		1		1
Health facility	−0.35(0.09)	0.71(0.59, 0.84)**	−0.02(0.08)	0.98(0.83, 1.14)
Media exposures				
No(ref)		1		1
Yes	−0.02(0.11)	0.98(0.80, 1.20)	−0.17(0.09)	0.84(0.71, 1.00)
Contraceptive use				
No(ref)		1		1
Yes	−0.11(0.08)	0.89(0.75, 1.06)	−0.015(0.07)	0.98(0.85, 1.14)
Post-natal check				
No(ref)		1		1
Yes	0.07(0.14)	1.07(0.83, 1.40)	0.014(0.11)	1.02(0.81, 1.27)
Number of children under five in the house hold				
0(ref)		1		1
1	0.07(1.71)	1.02 (0.61, 0.73)**	−1.23(0.56)	0.29(0.10, 1.08)
2	0.18(1.74)	1.19(1.05, 1.78)***	−0.97(0.56)	0.38(0.13, 1.14)
3	0.98(1.75)	2.67(2.47, 3.87)***	−0.76(0.57)	0.47(0.31, 1.42)
4 and above	0.57(1.77)	1.76(1.51, 3.15)***	−1.62(0.61)	0.19(0.06, 0.66)
Delivery by caesarean section				
Yes		1		1
No	0.51(0.19)	1.67(1.15, 2.40)**	0.19(1.17)	1.21(0.88, 1.67)
Community level mothers’ education				
Lower		1		1
Higher	−0.33(0.19)	0.71(0.49, 0.94)	0.30(0.15)	1.35(1.01, 1.81)
Community(zonal) wealth index				
Lower		1		1
Higher	−0.04(0.19)	0.94(0.66, 0.99)	−0.06(0.15)	0.92(0.70, 0.98)
Community(zonal) media exposure				
Lower		1		1
Higher	−0.04(0.22)	0.95(0.42, 0.99)	−0.06(0.17)	0.93(0.66, 0.97)
Community(zonal) antenatal visit				
Higher		1		
Lower	0.39(0.18)	1.48(1.02, 2.12) *	0.06(0.15)	1.06(0.79, 1.42)
Measure of dependency; OR (95% CI) = 8.5 (5.58, 18.7)*				

* = significant at 𝑎 = 0.05, ** = significant at 𝑎 = 0.01, and *** = significant at 𝑎 = 0.001.

At the community level, lower community education levels of mothers, community antenatal visits, community wealth index, and community media exposure significantly reduced the risk of HRFB of birth compared to higher levels. Lower community wealth index and community media exposure were also associated with a decreased likelihood of stunting compared to higher levels (Table 8).

## Discussion

This study aims to examine the association between high-risk fertility behavior of birth and stunting in under-five children based on the multilevel bivariate models in Ethiopia using the 2019 EMDHS. High-risk fertility is one of the obstacles to tackling child nutrition status in line with studies done in Asia, Africa, and Bangladesh (19, 20, 24). It is one of the deterring factors in the efforts put in by multiple parties to reduce the death of mothers and children. It is discovered that high-risk fertility has the power to affect the desired rate at which a given population has to grow, similar to studies in India, Nigeria, and Sub-Saharan (10, 16, 37). According to the findings of this study, 36.8% of children under the age of 5 years have stunted growth, and 60% of births fall into avoidable risk categories (HRFB) of birth. Approximately 1,177 (24%) children were stunted due to high-risk fertility behavior, in line with the report of EMDHS 2019 (28). From the categories of HRFB of birth, 21% of the risked group were under multiple risk categories, and 39% were grouped under single risk categories, which is in line with the study done in Ethiopia and Bangladesh (22, 28, 31, 45). Since the study is based on the association between HRFB of birth and SSC, high birth order was more significantly associated with stunting out of other components of HRFB of birth, which is similar to a study done in Bangladesh (22, 24, 33). A possible explanation for this association could be that higher-order births are more likely to be unwanted, which results in less attention and care for the child from parents: antenatal and postnatal care and child checkups decrease with the higher birth order; as a result, births of higher order might suffer from various health hazards as well as stunting. This study result is in line with the study done in Ethiopia, Bangladesh, and India (16, 24, 35, 38).

The finding of this study result demonstrated that around three-fourths of respondents were rural dwellers, and the highest prevalence of HRFB of birth and SSC occurred in rural residences, which account for 80.8 and 82.5%, respectively. The study also depicted that about 2,665 (54%) mothers had never attended school; among them, 68.1% of HRFB of birth and 60.7% of stunting were recorded, which is consistent with studies done in Ethiopia. Based on zonal aggregate value, 51% of mothers made health facility deliveries, and 44% had not yet received any formal education. This finding is in line with a study done in Ethiopia based on EDHS 2016 and Bangladesh (5, 20, 23, 33, 46).

Moreover, the study revealed that the odds of stunting increased as the age of children increased compared to age groups of 0–5 months. This finding was in agreement with the study done on the East Africa region (5). This demonstrated that starting other foods along with breastfeeding a child after 6 months of age increases the likelihood of taking polluted foods and minimizes the essential safety provided by breast milk. Additionally, as children mature, a high percentage of parents in rural regions fail to achieve their children’s optimal nutritional needs. This result is supported by the study done in Ethiopia (47). Similarly, the rate of being at high risk of birth also increased due to the short birth interval and birth order increment, which is similar to the study result of the pooled study done among nine East African countries (5).

Children from households in the poorest wealth index were also more likely to be HRFB of birth and stunted than children from the richest quintile, which is consistent with the findings of previous studies carried out in different developing countries (4). This could be because of the fact that increased income improves dietary diversity (39, 40), which in turn improves the adequacy of nutrient intake and nutritional status and underscores the importance of linking income-generating activities with other nutritional interventions. The findings indicated that children from Tigray regions were more stunted compared to children from Addis Ababa, which is similar to previous studies done in Ethiopia (13, 16, 29, 30) that compared regional variations in nutritional status. This difference may be from the differential nutritional intake, the difference in the environment, and geographical, socio-economic, and cultural differences. Women who attended primary and secondary school associated with a decreased probability of high-risk fertility behavior as compared to those who had no formal education. This finding was consistent with the study results in Ethiopia, India, and Bangladesh (6, 16, 18, 20, 33). This could be because those attending school had better knowledge and awareness about high-risk fertility behavior and a lower probability of experiencing early marriage. Moreover, children who were twins had a higher probability of being stunted. This may be due to insufficient breast milk, as well as infections due to incorrect formula feeding and scramble feeding. This finding is in line with the study results conducted in Ethiopia (25). Overall, the results of this study suggest that HRFB (older maternal age and shorter birth intervals) is significantly associated with childhood stunting. Concerning older maternal age, our findings revealed that children born to women who were older than 34 years had lower odds of being stinging as compared with those who had childbirth before age 34, including most recent delivery at an age less than 18 years. Thus, younger maternal age was a significant risk factor, while older maternal age was protective for childhood stunning. This finding is supported by the study that was done in the sub-African (32) region, which showed that younger age was significantly associated with higher stunning among children. The experience of younger women who become mothers and the limited resources at their disposal make it difficult for them to meet the nutritional requirements for their children, eventually exacerbating nutritional deficiencies that consequently affect childhood development. In addition, other predictors of stunting, such as the sex of the child being male, being resident of the Tigray region, and religion of Orthodox, Catholic, Muslim, and Protestant, are more likely stunted than others (4, 16, 27, 29, 48). Boys were more stunted than girls. Evidence of a higher risk in boys under the age of 5 would be faster growth (hence higher dietary requirements) in this year of life, and the other reasons may be hormonal and genetic factors. The result of this study also shows that mothers who had trouble accessing health services were often more likely to participate in high-risk fertility behaviors of birth. This study also demonstrated that the ANC visit of children decrease is more likely due to HRFB of birth and increased stunting status. This study finding is similar to the study finding of Tamirat et al. (5). This may be because women who had trouble accessing health services used less family planning and received less ANC care, resulting in shorter birth periods, births at an older age, and high birth orders. Those women who had no ANC follow-ups to the recent children were associated with increased risky fertility behaviors. During ANC, follow-up clinical checkups were made for the mother and fetus. Counseling about postnatal care included family planning choices for the widening of intervals between births. Thus, the low utilization of ANC during pregnancy might contribute to high-risk fertility behavior.

Furthermore, the findings of this study showed that the number of under-five children per household is a significant indicator of HRFB of birth for under-five children, which is in line with the study result of Tamirat et al. (5), Tessema and Tamirat (15), Rahman et al. (18), Naz et al. (19), Pedro et al. (20), and Teshale et al. (49). The possible reason may be that the birth interval between children was below the standard birth interval. The result also found that cesarean section delivery is associated with reduced risky fertility activity as compared to vaginal delivery. This finding is in line with the study done in Ethiopia, East Africa, and India (6, 20, 33, 49). This may be because frequent cesarean section deliveries reduced the number of pregnancies due to the possibility of negative effects of repeated cesarean section.

Among community (zone)-level variables, education level, media exposure wealth index, and antennal visit were more likely to reduce the risk of HRFB of birth in the same zones. This study finding is supported by the study findings of Tamirat et al. (5), Pedro et al. (20), Rahman et al. (23), Tessema et al. (33), and Howlader et al. (46). In addition, media exposure and higher wealth index were more likely to reduce the risk of stunting status of children than that lower and contrary to most of the previous studies, community education level were higher is more likely to increase the risk of stunting compared to lower community education level which is similar to the study finding of the study conducted in Ethiopia, East Africa, India, Bangladesh, Nepal, and Asia (5, 15, 18–20, 46). The reason might be that highly educated mothers have their own works and leave their children to other third parties such as care givers, and the children can get inadequate nutrition with a lack of breastfeeding. Moreover, it might be due to poor mother–child interaction between the child and educated mother that might trigger the occurrence of childhood stunting in the same zones.

From the findings of this study, interventions that target specific high-risk fertility behaviors without addressing underlying social determinants of health, such as wealth index, lack of education, or limited access to healthcare, may exacerbate existing health disparities. This can widen the gap between privileged and marginalized populations, perpetuating inequities in health outcomes. It is crucial for interventions aimed at reducing high-risk fertility behaviors to be implemented thoughtfully, with a deep understanding of the cultural context, community needs, and potential unintended consequences. This finding is in line with the study result of Mortazavi et al. (25), Ngandu et al. (27), and Woldeamanuel et al. (34).

## Conclusion

HRFB of birth encompasses behaviors deeply ingrained in the socio-cultural context of Ethiopian children, driven by traditional norms and values that promote high fertility rates. These practices, coupled with the high prevalence of child stunting, contribute to the need for closer birth intervals, early and late childbearing, and larger family sizes. To this end, the media-based reproductive health promotion campaigns should take into consideration variations in desire for children across regions, places of residence, and educational levels. Providing comprehensive culture-sensitive knowledge through well-designed and sustained media interventions is also required to raise the awareness of women and the community in general about the pros and cons of high-risk fertility and unreasonable panics associated with child deaths. Unjustified taboos about sex, pregnancy, birth, growth, and death have to be recursively and scientifically broadcasted through multiple media types. Hence, a total overhaul of media campaign approaches and strategies that can respond to specific but pervasive circumstances, needs, and challenges is also extremely important and a necessity of the time in Ethiopia. It is essential for the Ethiopian government and stakeholders to collaborate in efforts to prevent HRFB of birth and stunting, raise awareness, and provide community education to reduce high-risk fertility behavior.

The findings emphasize the urgency of reducing high-risk fertility, specifically by addressing patterns of too-early or too-late childbearing, high total birth numbers, and short birth intervals to mitigate the risk of chronic under nutrition in young children. These findings are also crucial in addressing maternal health and child health challenges in Ethiopia. As Ethiopia strives to meet Sustainable Development Goal 3, this study recommends that identified factors and population groups be given focus in fertility and child health programs. The study highlights the importance of understanding the causal relationship between HRFB of birth and children’s stunting status, as it is crucial for developing interventions aimed at improving children’s nutritional status. This has significant implications for public policy, particularly in terms of integrating family education and maternal and child health services. The finding of this study suggests that it is essential for the Ethiopian government and stakeholders to collaborate in efforts to prevent HRFB of birth and stunting, raise awareness, and provide community education to reduce high-risk fertility behavior. Furthermore, feature researcher investigating the causal link between high-risk fertility and children’s nutritional outcomes is necessary to address the public health priority of improving the nutritional status of children in Ethiopia. Moreover, it promotes collaboration between the Ethiopian government and stakeholders to implement awareness campaigns and educational programs for high-risk fertility behaviors and child stunting while also addressing healthcare access, nutrition, and social determinants of health through joint initiatives to be performed in the future to reduce the risk of high-risk fertility behavior of birth and child stunting.

### Limitation of study

Similar to other research studies, this study was not done without any limitations. There are certain limitations and challenges in this study, such as the lack of some variables and the presence of missing values in the Mini-Demographic and Health Surveys (MDHS), as well as reporting and recall biases. Moreover, this study did not identify any potential consequences that can happen both intentionally or unintentionally.

Those limitations will be solved through research findings and peer review by experts in the field to validate the methodology, analysis, and interpretation of results. Peer review can help identify potential biases, methodological flaws, and gaps in research design that need to be addressed. Handling missing data by statistical methodology such as multiple imputations, by implementing these strategies and addressing the limitations identified in previous studies, future research on high-risk fertility behaviors and child stunting in Ethiopia can be more robust, reliable, and informative for policymakers, practitioners, and stakeholders working to improve maternal and child health outcomes in the country.

## Data availability statement

The data analyzed in this study is subject to the following licenses/restrictions: The data will be available for reasonable request from the corresponding author. Requests to access these datasets should be directed to https://dhsprogram.com/data/EDHS/melkamu.ayana@gmail.com.

## Ethics statement

As the data used in this study was obtained from the secondary source of the Mini Ethiopian Demographic Health Survey (Mini EDHS 2019), verbal consent was not required from the study participants during data collection.

## Author contributions

MZ: Conceptualization, Data curation, Formal analysis, Investigation, Methodology, Software, Validation, Visualization, Writing – original draft, Writing – review & editing. WF: Conceptualization, Data curation, Formal analysis, Investigation, Methodology, Software, Validation, Visualization, Writing – original draft, Writing – review & editing.
